# The impact of slower walking speed on activities of daily living in patients with multiple sclerosis

**DOI:** 10.1111/ijcp.12003

**Published:** 2012-10-16

**Authors:** M Yildiz

**Affiliations:** Department of Neurology, Cantonal Hospital St. Gallen, St. GallenSwitzerland

## Abstract

**Aims:**

To identify the relevance and impact of walking speed (WS) over a short distance on activities of daily living (ADLs) in patients with multiple sclerosis (MS).

**Methods:**

An internet-administered survey of MS patients in four countries was distributed to 605 individuals in 2010. Participants had MS for > 5 years and must have reported difficulty walking as a result of MS. The impact of MS on walking and the effects of WS on ADLs were assessed based upon responses (scored on a scale of 1–10) to five questions and categorised *post hoc* as: high (8–10), moderate (4–7) or low (1–3) impact/importance.

**Results:**

Of the participants who completed the survey (*n* = 112), 60% were female patients, 63% were aged ≥ 45 years, and 55% had relapsing-remitting MS. Approximately, half of participants reported a high impact of MS on their general walking ability (46%) and their ability to increase WS over a short distance (55%). Up to 53% of participants reported avoiding ADLs because of concerns about WS; within this cohort, older male patients and patients with secondary-progressive MS were highly represented.

**Discussion:**

These results, which highlight the importance of WS to patients with MS and emphasise the impact of WS on health-related quality of life and ADLs, underscore the importance of clinical measures of WS, such as the timed 25-foot walk, in assessing walking in MS patients.

**Conclusion:**

Walking speed over a short distance has a significant impact on ADLs for patients with MS.

**What’s known**

Impaired mobility is a common feature of MS and can have a disabling effect on independence, employment, HRQoL and ADL. Walking impairment is estimated to affect 60% to > 90% of patients with MS (1–5) and is a progressive, worsening disability. Walking ability is perceived to have a greater impact on HRQoL than pain, dexterity or cognition. Therefore, mobility impairment is a major focus of clinical outcome measures for MS.

**What’s new**

The EDSS uses walking impairment in terms of distance walked to assess disability, but fails to appreciate the importance of walking speed. Knowledge of the specific impact of walking speed on ADLs from the patient’s perspective is limited. This article highlights the importance of walking speed on ADLs for patients with MS and emphasises the value of the T25FW as an assessment tool of walking impairment.

## Introduction

Multiple sclerosis (MS) is a chronic disabling disease of the central nervous system and is currently estimated to affect > 2 million people worldwide (6,7). Impaired mobility, defined as an activity limitation by the International Classification of Functioning, Disability and Health (ICF), is one of the most common and disabling features of MS and can have a profound effect on the independence, employment, health-related quality of life (HRQoL) and activities of daily living (ADL) (4,5,8–11) of affected individuals. Maintaining mobility was ranked as one of the highest priorities for patients with MS irrespective of the degree of impairment or duration of disease (4,12,13). Furthermore, walking was specifically and consistently ranked as the highest priority among 13 bodily functions (12). Walking impairment is estimated to affect 60% to > 90% of patients with MS (1–5) and can be progressive, resulting in worsening disability (3,14,15). Given that walking ability is perceived to have a greater impact on HRQoL than pain, dexterity or cognition (16), walking impairment is a major focus of clinical outcome measures for MS.

The most well-established and commonly used clinical assessments, the Expanded Disability Status Scale (EDSS) (17) score and the MS functional composite (MSFC) (18), use walking ability as a key measure of disability. The EDSS uses maximum walking distance and the need for walking aids to define a patient’s level of disability. For example, at EDSS 4.0, patients have limited walking ability, but are able to walk more than 500 m without aid or rest, whereas at EDSS 7.0, patients are able to walk no more than 5 m without rest even with aid (17). The timed 25-foot walk (T25FW) is one of three measures of the MSFC, which also assesses upper limb function using the 9-hole peg test and cognition using the paced auditory serial attention test (PASAT). The T25FW is the only validated, objective, specific measure of walking speed in patients with MS and can be used over a broad range of walking disabilities (19). Importantly, the T25FW correlates well with other measures of walking ability, including measures of distance (20–24). MS patients with walking and mobility problems have an increased burden of disease (16), reductions in HRQoL (16,25), a greater need for formal and informal care (26), loss of ability to work (26), increased healthcare utilisation (26) and higher costs associated with MS (10,26). Currently, knowledge of the specific impact of reduced walking speed on ADLs from the patient’s perspective is limited. A survey was therefore undertaken to identify the impact of MS on overall walking, the ability to increase walking speed (i.e. the ability to accelerate) and walking distance. Survey questions also assessed the relevance of walking speed over a short distance to ADL performance in patients with MS.

## Methods

Male and female patients with MS were recruited online from a sample of prescreened individuals willing to participate in online surveys using e-mail invitations. Respondents were surveyed between November 1–9, 2010 in France, Germany, Sweden and the United Kingdom (see Table 1 for survey questions). The survey was conducted via the internet with questions translated into the native language of each participating country. A total of 605 patients were invited to participate in the survey; the only criteria for inclusion was a self-reported diagnosis of MS for > 5 years and difficulty walking as a result of MS. Participants with an unconfirmed diagnosis of MS and those with no current or previous difficulty with walking as a result of MS were screened-out. Respondents reported general demographic and clinical information (e.g. age, MS course) and were asked five questions to assess the impact of their MS on walking ability and speed and the effects of their walking impairments on particular ADLs. Four questions were rated on a 10-point Likert scale ranging from 0 (no impact/importance) to 10 (extremely large impact/importance) and one question required a simple ‘Yes/No’ response. In question 2, patients were asked to assess their ability to increase their walking speed to determine their perception of the importance of acceleration vs. walking speed in general. Questions 4 and 5 were included to address the relevance of walking speed to ADL performance. The survey contained two additional questions that were not relevant to this study and, as such, were not included in this analysis. Respondents were rejected from the analysis if they completed the questionnaire in < 2 min, if they failed to press the keys associated with possible answers to any of the questions, or if responses did not meet the preset criteria for expected variability.

**Table 1:** Survey questions

Q1. How much of an impact has MS had on your walking ability generally?									
Q2. How much of an impact has MS had on your ability to speed up your walking pace (not running) over a short distance (e.g. when you need to cross the road)?									
Q3. How much of an impact has MS had on your ability to walk a longer distance (e.g. over 500 m?)									
Q4a. In your daily activities, how important is the ability to walk fast over a short distance in your house/flat (e.g. reaching the lavatory in time, going from one room to another, going to the front door)?									
Q4b. In your daily activities, how important is the ability to walk fast over a short distance outside your house/flat (e.g. to the bank, the shops, at work)?									

1	2	3	4	5	6	7	8	9	10

(extremely big impact)									(no impact)
Q5. Do you avoid any of the following activities because of concerns about your walking speed?									
Walking to your nearest shop				□ Yes □ No					
Cleaning your home				□ Yes □ No					
Crossing the street (e.g. at traffic lights)				□ Yes □ No					
Walking to the post box				□ Yes □ No					
Going to visit your neighbours				□ Yes □ No					
Other (please specify)				□ Yes □ No					
I don’t avoid any activities because of concerns about my walking speed □									

Responses to questions scored on the 1–10 scale were categorised *post hoc as*: high impact/importance (scores of 8–10), moderate impact/importance (scores of 4–7) or low impact/importance (scores of 1–3). Results of the survey and subgroup analyses were analysed using descriptive statistics and were collated for patient subgroups based on gender, age (< 45, 45–54 and ≥ 55 years) and MS course [relapsing-remitting (RRMS), secondary-progressive (SPMS), primary-progressive (PPMS)].

## Results

### Patient disposition

A total of 605 patients with MS were invited to participate in the study. Overall, 396 patients were screened-out because they did not have MS or did not experience difficulty with walking as a result of MS; an additional 51 patients who completed the questionnaire in < 2 min or failed to press the keys associated with possible answers to the questions or did not meet the criteria for expected variability were rejected. Those with missing demographic information (*n* = 37), or who did not complete all questions on the survey (*n* = 9), were also eliminated from the analyses to ensure quality of the results. Therefore, 112 met the inclusion criteria and adequately completed all five questions. Participant baseline demographic and clinical characteristics are summarised in Table 2. Overall, the study population was 60% female patients, 63% were aged ≥ 45 years, and 55% had RRMS, 33% had SPMS and 13% had PPMS (Table 2). Participants surveyed were approximately equally distributed between the four countries.

**Table 2:** Baseline demographic characteristics of survey participants

Baseline characteristics	Patients
Patients recruited, *n*	112
Patients participating in survey, *n*	112
Female, *n* (%)	67 (60)
**Age group (years), *n* (%)**	
< 45	42 (38)
45–54	42 (38)
≥ 55	28 (25)
**Country, *n* (%)**	
France	25 (22)
Germany	26 (23)
Sweden	28 (25)
United Kingdom	33 (30)
**MS course, *n* (%)**	
Relapsing-remitting	61 (55)
Secondary-progressive	37 (33)
Primary-progressive	14 (13)

### Impact of MS on walking ability, the ability to increase walking speed, and walking distance

**Figure 1:**
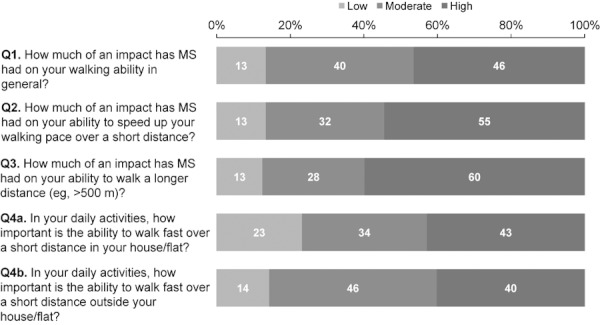
Categorical results for patients reporting low, moderate and high impact of MS on walking ability, speed and distance MS = multiple sclerosis. *On a scale of 0–10, where zero is of no impact/importance and 10 is of large impact/importance, low scores were 1–3, moderate scores were 4–7 and high scores were 8–10. Data are presented as % of patients (*n* = 112)

Nearly half of survey participants rated the impact of MS on their general walking ability question 1 (Q1) as high (score 8–10; Figure 1). Of these participants, a higher proportion was male patient, and more patients had SPMS than RRMS or PPMS(Q1; Table 3) In addition, older patients (age ≥ 55 years) were more likely to report a high impact of MS on their general walking ability than younger patients (age < 45 years or age 45–54 years, Q1; Table 3).

**Table 3:** Percentage of patients reporting a high impact (scores 8–10) of walking speed on ADLs by demographic and clinical characteristics

Subgroup	Q1	Q2	Q3	Q4a	Q4b
				
	*n* (%)	*n* (%)	*n* (%)	*n* (%)	*n* (%)
**Gender**					
Men	25 (56)	26 (58)	27 (60)	19 (42)	18 (40)
Women	27 (40)	35 (52)	40 (60)	29 (43)	27 (40)
**Age group (years)**					
< 45	19 (45)	22 (52)	22 (52)	20 (48)	18 (43)
45–54	16 (38)	19 (45)	25 (60)	15 (36)	12 (29)
≥ 55	17 (61)	20 (71)	21 (75)	13 (46)	15 (54)
**MS course**					
RRMS	22 (36)	29 (48)	33 (54)	25 (41)	25 (41)
SPMS	24 (65)	26 (70)	26 (70)	20 (54)	13 (35)
PPMS	6 (43)	6 (43)	8 (57)	3 (21)	7 (50)

ADL, activities of daily living; MS, multiple sclerosis; RRMS, relapsing-remitting multiple sclerosis; SPMS, secondary-progressive multiple sclerosis; PPMS, primary-progressive multiple sclerosis.					

Q1. How much of an impact has MS had on your walking ability generally?					
Q2. How much of an impact has MS had on your ability to speed up your walking pace (not running) over a short distance (e.g. when you need to cross the road)?					
Q3. How much of an impact has MS had on your ability to walk a longer distance (e.g. over 500 m?)					
Q4a. In your daily activities, how important is the ability to walk fast over a short distance in your house/flat (e.g. reaching the lavatory in time, going from one room to another, going to the front door)?					
Q4b. In your daily activities, how important is the ability to walk fast over a short distance outside your house/flat (e.g. to the bank, the shops, at work)?					

Over half of participants rated the impact of MS as high on their ability to increase their walking pace over short distances (Q2; Figure 1) or on their ability to walk longer distances (e.g. > 500 m; Q3; Figure 1). More patients with SPMS reported a high impact of MS on these aspects of walking ability (Q2 & Q3) than patients with RRMS or PPMS (Table 3). Slightly more men than women rated the impact of MS as high on their ability to increase their walking speed over a short distance (Q2; Table 3), and equal proportions of men and women rated the impact on their ability to walk distances > 500 m as high (Q3; Table 3). More than 70% of patients aged ≥ 55 years reported a high impact of MS on their ability to increase pace over short distances and ability to walk longer distances (Q2 & Q3; Table 3).

### Impact of walking speed on ADLs

The percentage of participants reporting a high impact of walking speed over a short distance on the ability to perform ADLs inside (Q4a) and outside (Q4b) the home was comparable (Figure 1). Similar proportions of men and women reported a high impact of walking speed on their ability to perform ADLs inside and outside the home (Table 3). More patients with SPMS reported a high impact of walking speed on their ability to perform ADLs inside the home, in comparison with those with RRMS or PPMS (Q4a; Table 3). However, for performing ADLs outside the home, more patients with PPMS reported a high impact of walking speed, in comparison with those with RRMS and SPMS (Q4b; Table 3). Interestingly, more younger (< 45 years) and older (≥ 55 years) patients reported a high impact of walking speed on their ability to perform ADLs inside and outside the home compared with patients age 45–54 years (Q4a and b; Table 3). Results for the Moderate and Low impact response categories are summarised in Figure 1.

**Figure 2:**
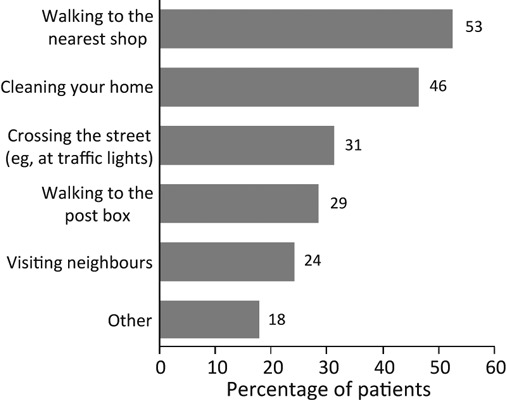
Q5: Do you avoid any of the following activities because of concerns about your walking speed? *Patients’ responses when asked the question ‘Do you avoid any of the following activities because of concerns about your walking speed?’ Data are represented as % of patients (*n* = 112)

In response to Q5, only 27% of patients did not avoid ADLs as a result of concerns about walking speed. When asked about specific ADLs, the activities avoided most were walking to the nearest shop, followed by cleaning the home (Figure 2). A higher percentage of patients with SPMS than those with RRMS or PPMS were represented in the cohort avoiding these particular ADLs (Q5; Table 4). This was also true for the cohorts that avoided crossing the street, walking to the post box or visiting neighbours (Q5; Table 4), activities that were avoided by over 20% of all respondents because of speed-related concerns (Figure 2). For all ADLs examined, more men than women reported avoiding activities; the activities of visiting neighbours, walking to the nearest shop or walking to the post box exhibited the largest gender bias (Table 4). Avoidance of four of the five ADLs assessed was more common in MS patients ≥ 55 years, whereas the tendency to avoid visiting neighbours was higher in the < 45 age group. Interestingly, younger patients (age < 45 years) avoided walking to the nearest shop, cleaning their homes, crossing the street or visiting their neighbours more than those in the 45–54 year category (Table 4).

**Table 4:** Percentage of patients reporting avoidance of six ADLs because of concern about walking speed by demographic and clinical characteristics

ADL	Gender		Age group			MS course		
		
	Men *n* (%)	Women *n* (%)	< 45 years *n* (%)	45–54 years *n* (%)	≥ 55 years *n* (%)	RRMS *n* (%)	SPMS *n* (%)	PPMS *n* (%)
Walking to the nearest shop	29 (64)	30 (45)	24 (57)	16 (38)	19 (68)	31 (51)	21 (57)	7 (50)
Cleaning your home	24 (53)	28 (42)	22 (52)	13 (31)	17 (61)	23 (38)	22 (60)	7 (50)
Crossing the street (e.g. at traffic lights)	18 (40)	17 (25)	14 (33)	10 (24)	11 (39)	20 (33)	9 (24)	6 (43)
Walking to the post box	18 (40)	14 (21)	9 (21)	11 (26)	12 (43)	15 (25)	12 (32)	5 (36)
Visiting neighbours	16 (36)	11 (16)	13 (40)	5 (12)	9 (32)	12 (20)	9 (24)	6 (43)
Other	4 (9)	16 (24)	2 (5)	14 (33)	4 (14)	7 (12)	9 (24)	4 (29)

ADL, activities of daily living; MS, multiple sclerosis; RRMS, relapsing-remitting multiple sclerosis; SPMS, secondary-progressive multiple sclerosis; PPMS, primary-progressive multiple sclerosis.

## Discussion

Almost invariably, patients with MS will experience difficulties with walking and mobility during the progressive, albeit heterogeneous course of the disease (1,3). Among those with walking impairments, a large percentage describes these difficulties as the most challenging aspect of their disease (2). Currently, there is limited information on the impact of mobility and walking impairments on ADLs in patients with MS (4). Previous studies have shown that general mobility loss (11) and objectively measured reductions in walking speed and distance (27) were associated with reduced ability to perform instrumental ADLs. This study evaluated the impact of walking speed over a short distance on patients’ ability to perform specific ADLs from the patient’s perspective.

In patients with MS, concern over walking speed restricted the ability to perform ADLs. It is estimated that more than 50% of patients with MS have limitations in ADLs and one-third are restricted in their ability to participate in social activities (28). Responses to question 5 also showed restrictions in ADLs and social activities and suggest that concerns about walking speed are a major contributor to this outcome. In another study of patients with MS, despite the high proportion of patients with mild disease severity, assessed by EDSS, walking disability and reduced ability to perform ADLs were reported in 43% and 48% of patients, respectively (29), which are comparable to the rates reported in this study. In a study of the ADLs and social activities of patients with MS living in Stockholm, it was reported that the most frequently affected ADLs were home cleaning and outdoor transportation (30). These items were also the most frequently affected in this study across all four countries, including Sweden (data not reported).

Notable gender- and age-related differences were observed in the reported impact of walking disability on ADLs. Higher proportions of men and older patients (age ≥ 55 years) reported avoiding activities because of concerns about their walking speed, compared with women and younger patients, respectively. A previous survey on patients’ perspectives of mobility impairment also reported a greater number of significant mobility difficulties in men than in women with MS (5). Although employment status was not assessed as part of this study, the reason for higher ADL avoidance among men may be work-related. Previous studies have indicated that men with MS are more likely to be employed than women, regardless of the severity of mobility impairment (11,31). This suggests that employed men may be more likely to feel the impact of their walking impairment on ADLs. However, gender has been shown to affect the prevalence, course and pathology of MS, in addition to the response to immunotherapy in multiple studies (32). Despite study outcomes often reporting conflicting gender-based results, in general, males have a later age of onset, more progressive disease and a more rapid progression of disease (32). Not surprisingly, more older patients report an impact of walking impairments on ADLs than younger patients, highlighting the progressive deterioration of walking ability in MS (14,33), while verifying that walking impairment is a concern for patients of all ages (4,12,13).

Patients with SPMS were more likely to rate the impact of MS as high on general walking ability, ability to speed up pace over a short distance and ability to walk longer distances than patients with other forms of MS. These findings are consistent with a previous study reporting that patients with SPMS have lower levels of physical activity compared with patients with RRMS (34). Natural history studies have indicated that nearly 50% of individuals with RRMS will progress to SPMS within 10 years (35) and ∼ 90% of patients initially diagnosed with RRMS develop SPMS within 25 years (36). Patients with SPMS not only report more walking-associated problems but also suffer the erosion of self-efficacy (i.e. the perception of capabilities for successfully executing a behaviour) for engaging in physical activity (34).

Concern over walking speed had a particularly high impact on ADLs, such as walking to the nearest shop, crossing the street and cleaning the home, making it difficult for patients with MS to execute these activities. In addition, walking speed has been shown to correlate with maximum walking distance (37,38). Taken together, these results highlight the importance of walking speed, and likely distance, on ADLs for patients with MS and emphasise the need for physicians to assess walking speed over short distances in their clinical routine. Improved walking speed is potentially linked to improved overall walking ability, along with increased distance and enhanced smoothness and balance. The T25FW is a validated, objective, specific measure of walking speed that can be used in the clinic (19) and is potentially the best clinical measure of walking ability correlated with the ability to perform ADLs (39). Moreover, the T25FW appears to be more sensitive to change when compared with the EDSS (40–42). Walking impairment has a high impact on a patient’s HRQoL (43), the well-being of their families (44) and the incremental costs for patients with MS (45). Therefore, patients with walking impairment may benefit from neurological rehabilitation, drug therapies and investment in healthcare services.

For the first time in 2010, the US Food and Drug Administration (FDA) approved dalfampridine extended release tablets to improve walking impairment in patients with MS. This approval was based on two phase 3 double-blind randomized clinical trials of prolonged-release fampridine 10 mg twice daily, in which walking speed (as assessed by the T25FW) was consistently increased in approximately one-third of patients with clinically definite MS of any disease course (46,47). Since then, prolonged-release fampridine (known as sustained- or modified release fampridine in some countries) has been approved in Europe, Australia, New Zealand and Canada.

Given that mobility impairment correlates positively with major depression in RRMS (48), the avoidance of physical activities as a result of walking impairment may be influenced by mood disorders. These are considered the most common neuropsychiatric disturbances in MS, with an estimated lifetime prevalence of 50% (49). A limitation of this survey is that depressive symptoms were not assessed, making it difficult to evaluate the contribution of mood to the avoidance of physical activities. Additional prospective research to explore the relationship between objective measures of walking speed and distance, and subjective assessments of the impact of these variables on patient HRQoL, particularly the ability to perform ADLs, is warranted.
